# Heart rate variability in patients with incomplete spinal cord injury during a single session of paired associative stimulation

**DOI:** 10.1038/s41598-025-25802-x

**Published:** 2025-11-25

**Authors:** P. Haakana, K. Holopainen, M. P. Tarvainen, A. Shulga

**Affiliations:** 1 BioMag Laboratory, Helsinki University Hospital and University of Helsinki, Helsinki, Finland; 2 Motion Laboratory, The New Children’s Hospital, Helsinki University Hospital and University of Helsinki, Helsinki, Finland; 3 Department of Technical Physics, University of Eastern Finland, Kuopio, Finland; 4 Department of Physical and Rehabilitation Medicine, Helsinki University Hospital and University of Helsinki, Helsinki, Finland

**Keywords:** Spinal cord injury, Paired associative stimulation, Transcranial magnetic stimulation, Heart rate variability, Parasympathetic activation

## Abstract

Heart-rate variability (HRV) reflects the balance between sympathetic and parasympathetic nervous systems and is commonly used as a biomarker for the state of the autonomous nervous system (ANS). Acute pain can also be detected by HRV. We applied a single session of paired-associative stimulation (high-PAS) combining high-intensity transcranial magnetic stimulation (TMS) and high-frequency peripheral-nerve stimulation (PNS) on patients with incomplete cervical spinal-cord injury (SCI). High-PAS has shown promising results in improving motor function after incomplete SCI. The cardiovascular effects of high-PAS have not been studied in patients with SCI. We used HRV to record any pain or stress-related reactions in patients with SCI and as an indicator for cardiovascular effects of high-PAS. Twelve patients with cervical SCI received a 20-min single session of high-PAS. HRV was recorded continuously from the 15-min rest period before the stimulation until the end of the 60-min follow-up period after the stimulation. Five-minute sections at the timepoints of PRE, STIM, POST, POST30, and POST60 min after the stimulation were analyzed. Although two patients reported uncomfortable sensations and one reported slight pain during the stimulation, no signs of increase in sympathetic nervous system activation were detected. Instead, we observed moderate parasympathetic activation occurring during the high-PAS, similar to our previous findings in healthy participants.

**Supplementary Information:**

The online version contains supplementary material available at 10.1038/s41598-025-25802-x.

## Introduction

Spinal-cord injury (SCI) alters both motor and sensory functions and significantly disrupts the autonomic nervous system (ANS) and cardiovascular regulation. Severe injuries occurring at or above the T6 level that damage descending pathways from the brain to the sympathetic preganglionic neurons can disrupt supraspinal sympathetic control^1^, resulting in decreased sympathetic activity^2^. Parasympathetic regulation of the cardiovascular system, mediated by the vagus nerve, remains intact after SCI^2^. Autonomic dysreflexia (AD), a major cardiovascular complication in SCI, is observed more frequently in motor complete injury (American Spinal Cord Injury Association [ASIA] impairment scale [AIS] A or B)^3,4^. AD is characterized by systolic blood pressure increase > 20 mmHg above baseline in response to noxious stimuli below the injury level^5^.

A non-invasive neuromodulation, high-PAS, combines high-intensity transcranial magnetic stimulation (TMS) and high-frequency peripheral-nerve stimulation (PNS). This protocol is individualized by measuring patient-specific motor-evoked potential (MEP) and F-response latencies^6,7^, ensuring that both stimulation-induced volleys arrive in synchrony to corticomotorneuronal synapses in the corticospinal tract^6^. High-PAS enhances motor capabilities after incomplete chronic SCI^7–9^ by increasing voluntary activation^10^. This improvement is noted as an increase in Manual Muscle Testing^11^ scores and better performance in functional tasks, including handgrip, Box and Block test, and walking speed^7^.

The effects of high-PAS on cardiovascular function remain largely unexplored. As high-PAS requires high-intensity TMS and high-frequency peripheral stimulation applied over several weeks or months, it is important to understand its possible effects on autonomic functions and pain. Patients aged between 17 and 75 years and otherwise healthy besides SCI were studied in our previous experiments^7^. The elderly population has an increased incidence of SCI^12^, which leads to challenges related to comorbidities during rehabilitation. Thus, evaluating cardiovascular safety of high-PAS is important.

Heart-rate variability (HRV) is used as a biomarker of autonomic nervous system function. It reflects the dynamic balance between sympathetic and parasympathetic activity. HRV is derived from fluctuations in the time intervals between heartbeats and can be analyzed in time, frequency, and nonlinear domains^13^. In time-domain analysis, heart rate (HR) over time and length of intervals between normal-to-normal (NN) beats are assessed. In contrast, frequency domain analysis evaluates power distribution across different frequency ranges using a Fast Fourier Transform (FFT) algorithm^13^. Reduced HRV is associated with increased mortality risk^14^. Pain is perceived as a subjective experience related to actual or potential tissue damage^15^ and systems regulating pain perception interact with autonomic control. Thus, HRV can reflect how the autonomic nervous system reacts to nociceptive stimulation^15,16^. Reduced HRV is also observed with anxiety^17,18^ and psychological stress^19,20^, and these results have been linked to validated subjective instruments to evaluate these conditions. Notably, reduced HRV is also observed in patients with chronic pain^15^ and in some people with SCI^21^. Consequently, we utilize HRV as a biomarker for assessing cardiovascular safety of high-PAS, monitoring the balance between sympathetic and parasympathetic nervous systems.

Reduced HRV is often observed after high-level SCI. Diminished low frequency (LF) power, total power, and low frequency over high frequency (LF/HF) ratio is observed in high-level SCI compared with those with lower-level injuries^22^ and healthy controls^23,24^. Additionally, patients with SCI have lower arterial elasticity than healthy controls, which may induce a higher load for cardiac function^25^. Furthermore, some patients with SCI present with reduced standard deviation of NN (SDNN) and impaired diastolic orthostatic blood pressure responses during orthostatic challenges compared with healthy individuals, along with a higher variability in blood pressure^24^. Circulatory^26^ and cardiovascular diseases are one of leading causes of mortality in the SCI population^27^. One study has used HRV, specifically SDNN, to predict the onset of AD, presented as a pattern of initial decrease followed by a sharp increase^28^.

Different neuromodulation methods may influence HRV, in addition to enhancing motor recovery after SCI. Anodal transcranial direct current stimulation (tDCS) increases sympathetic activity as measured by increase in LF in healthy participants^29^ and in patients with SCI^30^ when targeted to the primary motor cortex. The stimulation was applied for 15 min at an intensity of 1 mA for healthy participants^29^ and 12 min at 2 mA for patients with SCI^30^. In contrast, peripheral electrical stimulation with paced breathing may enhance parasympathetic tone^31^. Although the effects of spinal-cord stimulation on HRV are inconclusive^32^, cardiovascular-focused parameters of the applications have demonstrated improved HRV^33^. Overall, HRV has potential as a biomarker for understanding the autonomic effects of neuromodulation and for monitoring treatment efficacy.

Recently, we reported no increase in sympathetic activation during high-PAS in healthy individuals^34^. The aim of this study was to investigate the effects of a 20-min high-PAS session on HRV in individuals with incomplete SCI and to confirm that high-PAS does not increase sympathetic activation related to pain or stress. The applied protocol was previously validated in healthy participants^34^.

## Methods

### Participants

To determine a suitable sample size, power analysis with Gpower 3.1.9.7 software was calculated from our pilot data. The minimum sample size needed was 8 for HF power with medium-to-large effect size using Cohen’s criteria (Cohen 1988), significance level at α = 0.05, effect size = 1.6, and power = 0.95. Fourteen patients with incomplete cervical SCI were recruited. Data from 12 participants (10 males, mean age 56.5 ± 11.5 years) were included in the final analysis. Time since injury was (mean ± SD) 6.4 ± 4.1 years. Ten patients had grade D on AIS^35^ and 2 patients had grade B. All participants were injured at cervical level. The patients had previous experience with high-PAS, and the time since last active high-PAS session was at least 5 months. Written informed consent was obtained from each participant prior to the study. All experiments were performed in accordance with the declaration of Helsinki. The Helsinki University Hospital Regional Committee on Medical Research approved the study (HUS/1280/2016). Exclusion criteria were any brain pathology, implemented devices, cardiac diseases, other neurological diseases except SCI, and pregnancy. Detailed information on patient background is presented in Table 1.

**Table 1:** Background information of patients.

ID	ASIA	Time since injury (years)	NLI	Etiology	CNS-active medication	Other medication	Smoking/other nicotine product intake
1	D	2	C3	Traumatic	Baclofen, Pregabalin		n/a
2	D	8	C1	Non-traumatic	Gabapentin, Duloxetine	Losartan/hydrochlorotiazide for high BP	no
3	D	11	C4	Non-traumatic	Amitriptyline 30 mg	Bisoprolol 2.5 mg for high BP	n/a
4	D	5	C1	Non-traumatic	Buprenorphine 5 μg/h, Duloxetine 90 mg		no
5	D	7	C2	Non-traumatic	Pregabalin 225 mg, Baclofen 50 mg		n/a
6	B	12	C7	Traumatic	Tramadol 100 mg + 50 mg, Baclofen 25 mg		n/a
7	D	8	C5	Traumatic	Baclofen 60 mg, Tizanidine 24 mg, Pregabalin 225 mg	Bisoprolol 2.5 mg for high BP	n/a
8	D	2	C1	Traumatic	Amlodipin 5 mg, Baclofen 5 mg × 3, Gabapentin 300 mg		Yes
9	D	3	C3	Traumatic	Amlodipin 5 mg, Baclofen 5 mg × 3		Yes
10	D	12	C1	Non-traumatic	Buprenorphine 0.5 mg on demand, Baclofen 5 mg	Beclometasone + formoterol + glycopyrrolate for asthma	Yes
11	B	7	C7	Traumatic	None	Aspirin for thrombosis prevention	Yes
12	D	2	C5	Traumatic	Baclofen 10 mg 2 × /day, Pregabalin	Lerkanidipin 10 mg/losartan 12.5 mg for high BP	No

*ASIA* American Spinal Cord Injury Association, *NLI* neurological level of injury, *CNS* central nervous system, *BP* blood pressure, *n/a* no answer.

### Experimental setup

**Fig. 1:**
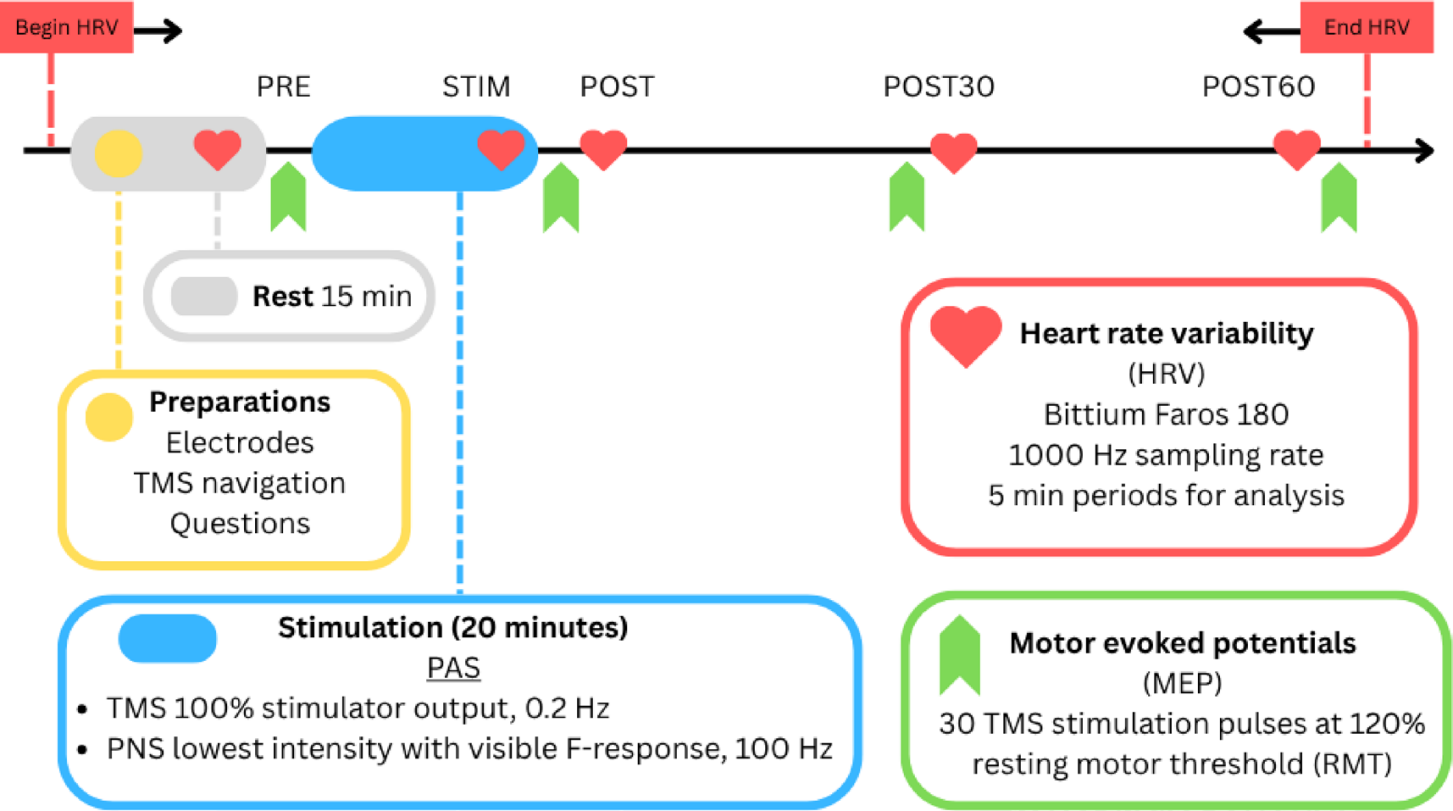
Protocol flowchart for high-PAS sessions. TMS = transcranial magnetic stimulation, *HRV* heart-rate variability, *PAS* paired-associative stimulation, *MEP* motor-evoked potential, and *RMT* resting motor threshold.

The experiments were performed at the BioMag laboratory at Helsinki University Hospital (Helsinki, Finland). One session lasted up to 120 min, including 20-min active stimulation and 60-min follow up (Fig. 1). Individual stimulation parameters were determined on a different day than the actual stimulation. Prior to the stimulation, participants were seated for 15 min to minimize the effect of commuting on HR, blood pressure, and HRV. Participants were asked to avoid alcohol intake and physical stress for at least 24 h and caffeine intake for minimum 6 h prior to the stimulation; use of nicotine products was not controlled. At the beginning of the session, self-adhesive electrodes (Blue sensor, Ambu A/S, Ballenrup, Denmark) were attached for electrocardiogram (ECG) monitoring of HRV and for electromyogram (EMG, Neuroline 720, AMBU A/S, Ballerup, Denmark) recordings of MEPs. Patients were asked about potential HRV-influencing factors (supplementary data Table 1). A cuff for blood pressure measurements (M6 AC, Omron, Kyoto, Japan) was attached to the left upper arm.

Participants were seated in a semi-seated position in the TMS chair and instructed to relax and breathe normally throughout the session. A ROHO® anti-pressure sore cushion was used to reduce the risk of pressure ulcers. Talking and phone use were not permitted. During the stimulation, participants were instructed to think about moving their thumb (motor imagery), similar to our previous study with healthy participants^7,36^. During the 60-min follow up, light activities such as talking, using their mobile phone, and regular changes in sitting position were allowed, but getting up from the chair was not recommended.

### Stimulation parameters

A Nexstim eXimia magnetic stimulator system (Nexstim Ltd, Helsinki, Finland) with a figure-of-eight coil with outer diameter 70 mm was used for navigated TMS (nTMS). Prior to stimulations, a 3T head magnetic resonance imaging scan (MRI, Magnetom Skyra 3, Siemens AG, Erlangen, Germany) was obtained for all participants. Three-dimensional (3D) images with T1 and T2 sagittal and coronal reconstruction were scanned. The images were imported into the TMS software for navigation. The TMS system reconstructed a 3D head model based on the MRI image and anatomical landmarks were manually registered to their corresponding locations on each participant’s head at the beginning of each session using the 3D navigation system.

The target area (hotspot) for the abductor pollicis brevis (APB) muscle in the primary motor cortex (M1) was determined using individual MRI images and EMG. The hotspot was the location where TMS elicited the largest MEPs recorded on APB. The navigated TMS system enabled consistent localization and use of the same hotspot for TMS and MEP recordings throughout the study. MEPs were recorded with an EMG device integrated to the stimulator (band-pass filter 10–500 Hz, sampling rate 3 kHz) and surface electrodes placed over the APB of the self-reported dominant hand (right n = 12, left n = 2). The resting motor threshold (RMT) of the hotspot was determined as the lowest TMS intensity that produced MEPs of at least 50 μV in a minimum of 5/10 attempts^37^. Individual intensity of 120% of RMT was used for MEP recordings during experiments (Table 2). For MEP latency determination, 15 MEPs were recorded using 100% of maximum stimulator output and their mean value was calculated. For PNS, similar electrodes as those used for EMG were placed over the median nerve. For F-latency determination, 0.2-ms pulses at supramaximal intensity were applied and a minimum latency for F-responses from APB was determined^38^. Stimulation intensity was set individually to the lowest intensity where F-responses were detectable when recorded with a 1-ms stimulation^39^. The interstimulus interval (ISI) between TMS pulse and the first pulse of the PNS pulse train was calculated individually applying the formula [F latency—MEP latency]^40^. MEPs were recorded before (PRE), during (STIM), immediately after (POST), 30 min after (POST30), and 60 min after (POST60) high-PAS.

**Table 2:** Stimulation parameters.

ID	Peripheral stimulation		Cortical stimulation			ISI = F-MEP (ms)
	F latency (ms)	Stimulation intensity (mA)	RMT %SO	120% RMT intensity	MEP latency (ms)	
1	32.6	5	96	> 100	28.4	4
2	30.5	3.5	58	70	23.4	7
3	27.8	5.5	44	53	26.9	1
4	26.3	3	40	48	21.1	5
5	34.2	5	53	64	25.0	9
6	31.3	5.5	64	77	24.3	7
7	30.6	2.5	62	74	27.1	3
8	30.8	2.5	72	86	33.1	− 2
9	30.5	9	58	70	25.4	5
10	32.9	2.5	53	64	30.8	2
11	25.3	10*	48	58	28.5	− 3
12	28	2.5	55	66	25.4	3

*RMT* resting motor threshold, *SO* stimulator output, *MEP* motor-evoked potential, *ISI* interstimulus interval. * highest tolerated intensity.

### Paired-associative stimulation

We applied high-PAS as previously described in detail^6,7^. Navigated TMS was applied with single pulses at 100% of the maximum stimulator output (MSO). PNS (Dantec Keypoint® Natus Medical Incorporated, California, USA) was delivered as trains of 6 pulses, 1-ms biphasic square pulses at 100 Hz to the median nerve in the middle of the palmar side of the wrist. Presentation® software (Neurobehavioral Systems Inc., Albany, USA) was used to synchronize TMS and PNS pulses to be delivered with individual ISIs once every 5 s. Altogether 240 PAS sequences were given during the 20-min stimulation.

### Heart rate variability

HRV was measured with Bittium Faros 180 (Bittium ltd, Oulu Finland) device with 3 ECG electrode configuration with sampling rate 1000 Hz. The ECG electrodes and recording device were attached at the beginning of the session and recording continued until removal. The following 5-min periods were used for HRV analysis: at the end of the 15-min rest period prior to stimulation (PRE), at the second half of 20-min stimulation period (STIM), immediately after post-stimulation MEP measurement (POST), right after post 30 (POST30), and right before or after post 60 (POST60) MEP measurement (Fig. 1). A representative noise-free 5-min segment was selected for the HRV analysis in agreement by two investigators. Thus, the timing of the selected segments might vary by minutes across patients in relation to the protocol. HRV data were analyzed with Kubios HRV Premium software (Kubios ltd, Kuopio Finland). On average, the software used beats correction on 0.3% of the data (maximum 2.2%). Analyzed HRV variables are shown in Table 3. LF band used for the analysis was 0.04–0.15 Hz. Room temperature was kept at 20–22°C and neutral indoor light was used.

**Table 3:** Description of adopted time-domain, frequency-domain, and nonlinear HRV parameters and their main association to autonomic nervous system function. ^34^. Modified from

HRV parameter	(units)	Description	Association to ANS function
*Heart-rate parameters*			
Mean RR	(ms)	Mean of the selected beat-to-beat RR interval series, inversely proportional to mean heart rate	PNS↑ and SNS↓
Mean HR	(bpm)	Mean heart rate, inversely proportional to mean RR	SNS↑ and PNS↓
SD HR	(bpm)	Standard deviation of heart-rate beats	PNS↑
Max HR	(bpm)	Maximum heart rate (evaluated as 5-beat average)	SNS↑ and PNS↓
Min HR	(bpm)	Minimum heart rate (evaluated as 5-beat average)	SNS↑ and PNS↓
*Time-domain parameters*			
SDNN	(ms)	Standard deviation of all normal RR intervals (normal-to-normal intervals, NN), demonstrating overall variability	PNS↑ and SNS↕
RMSSD	(ms)	Root mean square of successive differences between RR intervals, demonstrating beat-to-beat variation	PNS↑
NN50	(beats)	Number of consecutive NN interval pairs differing more than 50 ms	PNS↑
pNN50	(%)	NN50 divided by the total number of all NN intervals, demonstrating beat-to-beat variation	PNS↑
*Frequency-domain parameters*			
LF power	(ms^2^)	Low frequency (LF) power (frequency range 0.04–0.15 Hz) extracted from RR interval time series power spectrum	SNS↕
LF power	(n.u.)	LF power in normalized units (n.u.) representing the relative power in proportion to total power (TP) minus VLF power: LF [n.u.] = LF power [ms^2^] / (TP [ms^2^]—VLF power [ms^2^])	SNS vs. PNS
HF power	(ms^2^)	High-frequency power (frequency range 0.15–0.5 Hz) (synchronous with respiration); estimates parasympathetic/vagal activation	PNS↑
HF power	(n.u.)	HF power in normalized units (n.u.) representing the relative power in proportion to TP minus VLF power: HF [n.u.] = HF power [ms^2^] / (TP [ms^2^]—VLF power [ms^2^])	PNS vs. SNS
LF/HF		LF/HF power ratio	SNS vs. PNS
Resp	(Hz)	Respiratory rate estimated from the ECG and HRV data	
*Nonlinear parameters*			
Poincaré SD1	(ms)	In Poincaré plot, the standard deviation of RR intervals perpendicular to (SD1, demonstrating beat-to-beat variability) the line of identity	PNS↑
Poincaré SD2	(ms)	In Poincaré plot, the standard deviation of RR intervals along (SD2, demonstrating overall variability) the line of identity	PNS↑ and SNS↕
SD2/SD1		SD2/SD1 ratio	SNS vs. PNS

*SNS* sympathetic nervous system, *PNS* parasympathetic nervous system, ↑ indicated ANS activation tends to increase the HRV parameter, ↓ indicated ANS activation tends to decrease the HRV parameter, ↕ diverse association with ANS function, indicated ANS activation may increase or decrease the HRV parameter.

### Statistical analysis

IBM SPSS 27 software was used for statistical analysis. Nonparametric tests were used due to the small number of participants. Differences within session were assessed with Friedman’s ANOVA. Dunn-Bonferroni was used for pairwise comparisons. Effect sizes are reported as Kendall’s W for Friedman test and % change for pairwise comparisons. For MEP, changes were compared between PRE and POST, POST30, and POST60. For HRV variables, pairwise comparisons were applied between every timepoint.

## Results

All participants completed the study. Data from 2 patients were excluded due to noise in HRV measurements. Data from 12 patients were selected for analysis. No adverse events were observed. One timepoint (POST30) from 1 patient was removed due to low breathing frequency (0.11 Hz).

**Fig. 2:**
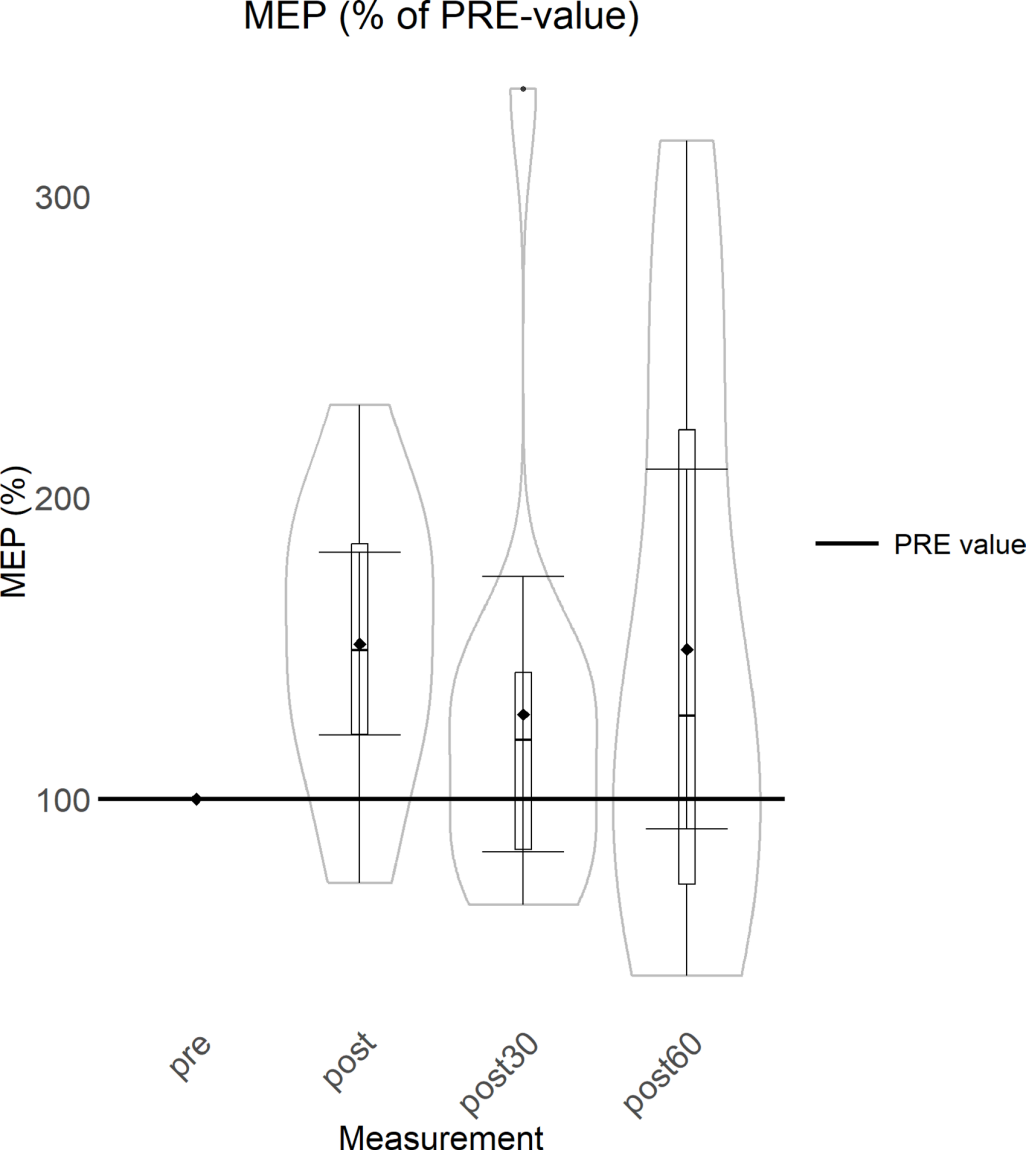
MEP data (% of PRE value) presented as violin plots showing data distribution. Boxplots indicate interquartile range and median and diamonds indicate group means with error bars representing 95% confidence intervals.

There was a non-significant (p = 0.231) trend of increase from PRE to POST (51%) MEP amplitude (Fig. 2), and the trend remained elevated from PRE level at POST30 (28%) and POST60 (49%) timepoints.

**Fig. 3:**
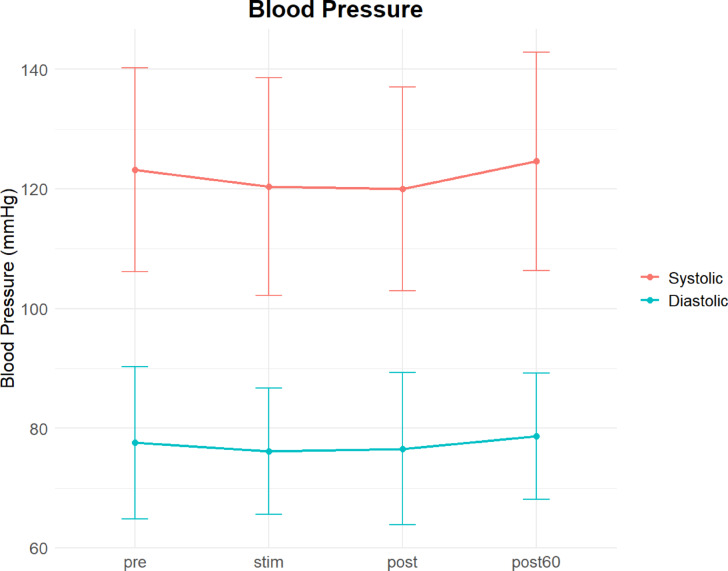
Mean ± SD of blood pressure measured before stimulation (PRE), in the middle of the stimulation (STIM), after post-stimulation MEPs (POST), and before the last MEPs (POST60). There were no significant changes between timepoints (*p* = 0.900) for systolic blood pressure or (*p* = 0.311) for diastolic blood pressure.

Blood pressure (Fig. 3) did not change (*p*= 0.090) during or after high-PAS. At the individual level, mean fluctuations between time points did not exceed ± 16 mmHg.

HR variables decreased throughout the recording. Maximum HR decreased at STIM (*p* = 0.008) and minimum HR at POST30 (*p* = 0.005) and POST60 (*p* = 0.045). The changes in mean RR were not significant. Results are presented in supplementary data. There were no significant changes in mean HR. On a group level, there was a decreasing trend during the stimulation (70 to 68 bpm). Five patients had asymptomatic bradycardia (HR < 60 bpm); one patient had HR 62 bpm at PRE that decreased to 58 during the stimulation. SD HR decreased during stimulation (− 21%) when compared with PRE and increased at POST (*p* = 0.001) and POST30 (*p* = 0.005) when compared with STIM values.

**Fig. 4:**
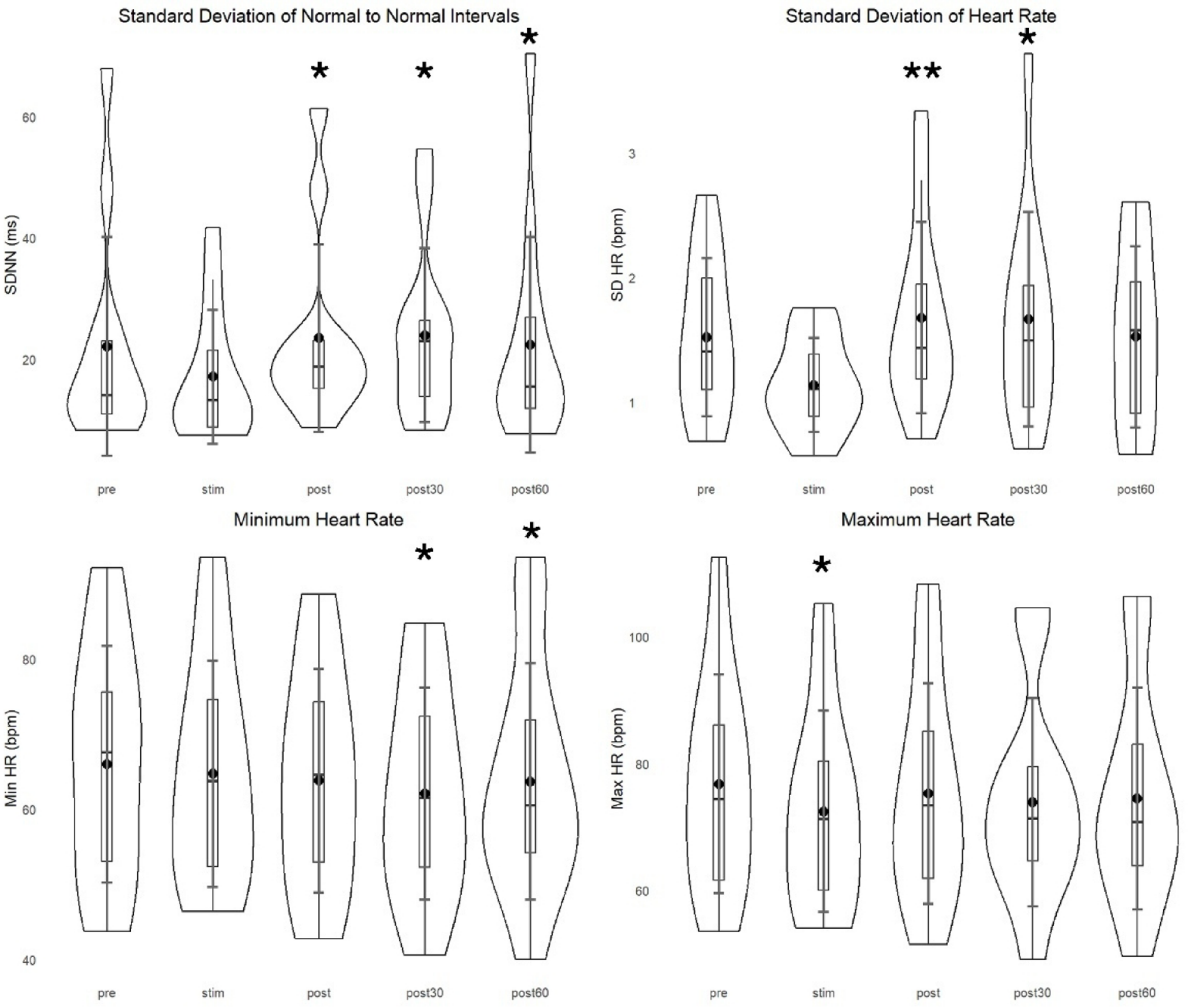
Standard deviation for normal-to-normal intervals revealed a significant increase when compared with STIM at POST (*p* = 0.030), POST30 (*p* = 0.012), and POST60 (*p* = 0.045). Similarly, the standard deviation of heart rate increased at POST (*p* = 0.001) and POST30 (*p* = 0.030) when compared with STIM. Heart-rate variables showed a steady decreasing trend in minimum heart rate at POST30 (*p* = 0.005) and POST60 (*p* = 0.045) and for maximum heart rate at STIM (*p* = 0.008) when compared with PRE. * *p* < 0.05, ** *p* < 0.005. Violin plots represent data distribution, boxplots indicate interquartile range and median, and diamonds show group means with error bars representing 95% confidence intervals.

In time domain, NN50 and pNN50 were not analyzed due to low HRV in some participants; less than half of the patients had no results for these variables. When compared with PRE, SDNN initially had a decreasing trend during stimulation (− 16%) and then increased at POST (*p* = 0.030), POST30 (*p* = 0.012), and POST60 (*p* = 0.045) when compared with STIM. RMSSD was not significant. Selected HR variables and time-domain variables are presented in Fig. 4.

In frequency domain, the results were consistent with our previous work in healthy participants^34^. LF power (both ms^2^ and n.u.) increased from STIM to POST (ms^2^, *p* = 0.001 and n.u., *p* = 0.002), POST30 (*p* = 0.008 and *p* = 0.005, respectively), and POST60 (*p* = 0.019 and *p* = 0.030, respectively). Additionally, LF (n.u.) decreased from PRE to STIM (*p* = 0.008). HF power (n.u.) increased during STIM when compared with PRE (*p* = 0.008), POST (*p* = 0.002), POST30 (*p* = 0.005), and POST60 (*p* = 0.030). HF power (ms^2^) change was not significant. Breathing frequency decreased from PRE to POST (*p* = 0.019). LF/HF ratio had a similar pattern to LF power with a decrease from PRE to STIM (*p* = 0.008) followed by an increase from STIM to POST (*p* = 0.002), POST30 (*p* = 0.005), and POST60 (*p* = 0.030). Comparisons with other timepoints were not significant.

**Fig. 5:**
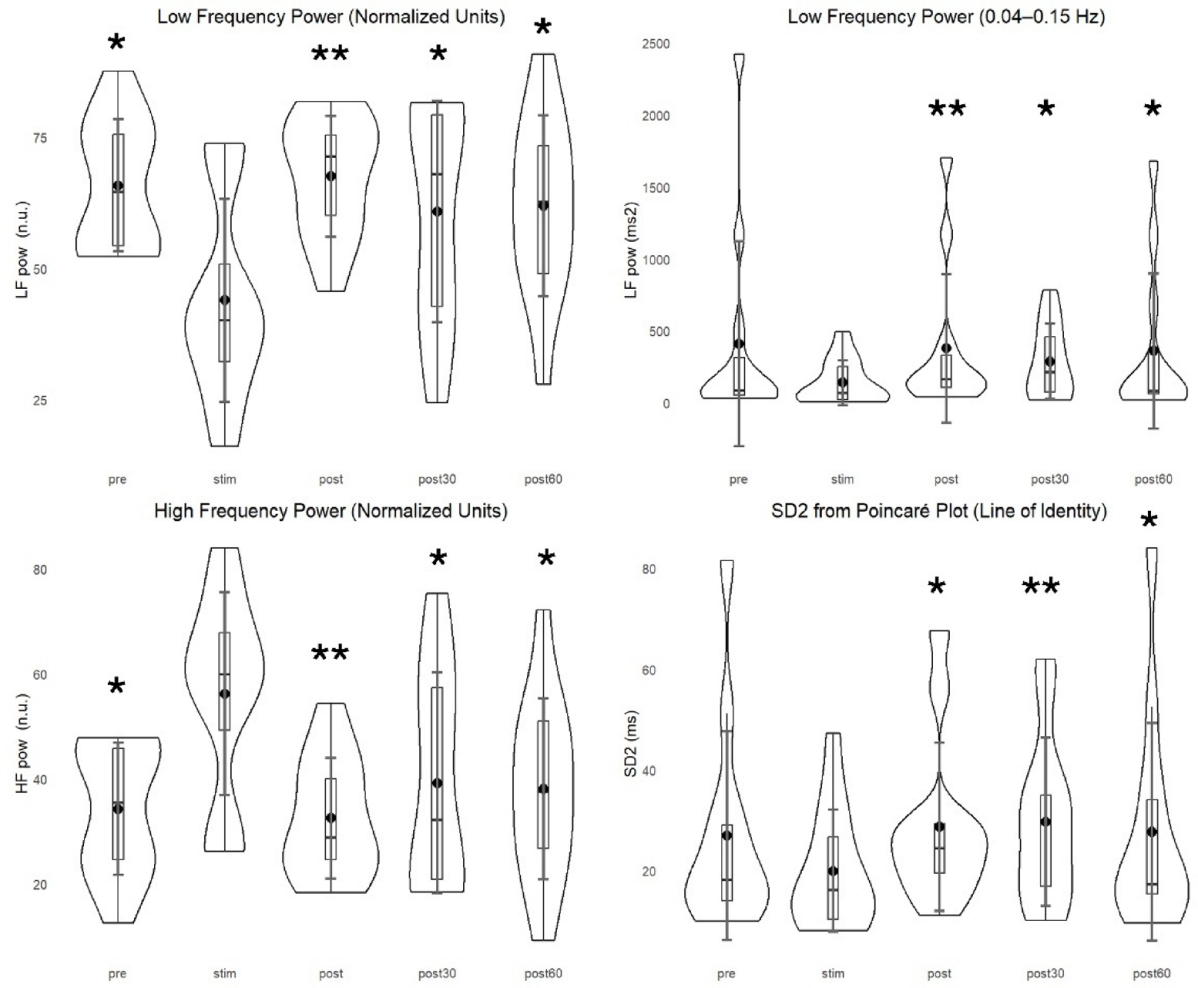
Frequency-domain analysis showed a significant decrease in LF power (n.u.) and an increase in HF power (n.u.) during STIM when compared with PRE (*p* = 0.008), POST (*p* = 0.002), and POST30 (*p* = 0.005). * *p* < 0.05, ** *p* < 0.005. Low-frequency power (ms^2^) increased from STIM to POST (*p* = 0.001), STIM to POST30 (*p* = 0.008), and from STIM to POST60 (*p* = 0.019). Violin plots represent data distribution, boxplots indicate interquartile range and median, and diamonds show group means with error bars representing 95% confidence intervals.

Non-linear HRV variables increased in SD2 from STIM to POST (*p* = 0.005), POST30 (*p* = 0.002), and POST60 (*p* = 0.012). SD1 change was not significant. SD2/SD1 ratio increased from STIM to POST (*p* = 0.030). Frequency domain and non-linear HRV variables are presented in Fig. 5.

Tiredness increased slightly during the stimulation (average from 2.08 to 2.75, *p* = 0.033, on a scale 1–5 where higher score indicates increased tiredness). This is consistent with our previous study reporting alertness during high-PAS^36^. One patient reported pain during high-PAS. Half of the patients (6) found high-PAS neutral. Two patients found high-PAS uncomfortable and four patients found it comfortable or very comfortable.

## Discussion

A single session of 20-min high-PAS did not induce an increase in sympathetic activation in patients with incomplete cervical SCI. Instead, average parasympathetic activation increased, LF power decreased, HF power increased, and Poincare plot SD2 increased after high-PAS. For HR variables, maximum and minimum HR decreased from PRE, max HR decreased during the stimulation, and min HR decreased at POST30 and POST60. Additionally, the standard deviation for HR increased after stimulation at POST and POST30. SDNN continued to increase at POST60. These results are consistent with our previous observations in healthy participants. Thus, high-PAS did not cause discomfort that could be observed as a change in HRV.

In this study, NN50 and pNN50 values were omitted from the analysis due to a low number of successive intervals, which is related to a lower HRV. Consequently, this weakens the specificity of the analysis of parasympathetic activity in this study. Lower HRV was expected as this has been reported in patients with SCI^1^, especially those with cervical-level injuries. Patients had lower HRV than healthy participants in our previous study and SDNN and RMSSD values were smaller in patients than in healthy individuals (SDNN average 22.1 ms and 50 ms and RMSSD 21.7 ms and 43.8 ms, respectively)^34^.

HRV has not been previously analyzed during high-PAS in patients with SCI. However, we have reported HRV responses to high-PAS in healthy participants^34^. Low-frequency (< 1 Hz) TMS studies revealed reduced HR and high-frequency (> 1 Hz) TMS protocols increased RMSSD and decreased LF/HF ratio^41^. Both TMS and sham stimulation increased HF in one stud^42^, consistent with our findings.

An increased baroreflex activity, observed as increase in LF/HF ratio and LF power occurred during anodal tDCS^29^. However, other stimulation studies on high density (HD)-tDCS revealed reduced HR and an increase in LF/HF ratio^43^ and increase in SDNN and LF power (ln)^44^ after stimulation, but not after a sham experiment in healthy participants. Similar results have also been observed during tDCS in patients with SCI; increased LF power and LF/HF ratio and decreased HF power have been observed^30^. However, tDCS differs substantially from navigated TMS in neuronal activation and focality of the stimulation^45^.

The autonomic nervous system regulates cardiovascular activity through neurotransmitters and neuroimmune processes, and parasympathetic activation is controlled through postganglionic fibers^46^. Several brain regions might be involved in ANS activity, indicating that brain stimulation might have potential for modulation of autonomic nervous system^45^. Effects of neuromodulation on HRV have not been extensively studied and many experiments lack proper randomization protocols, but both TMS and tDCS may alter HRV depending on the stimulation area^45^. Although high-frequency median nerve stimulation induces an increase in sympathetic nervous system activation^47^, combining high-frequency PNS with low-frequency TMS stimulation did not produce increased sympathetic nervous system activation in this study.

High-PAS effects most likely do not reach cardiovascular control. As concluded in our previous study,^34^ it is plausible that sitting quiet for 20 min increases parasympathetic activity in patients during high-PAS. There were individual differences in HRV variables, in addition to frequency domain variables, where all participants had LF decrease and HF increase.

Only a single patient had no medication. The potential influence of prescribed medications on our results, particularly in instances of polypharmacy, cannot be excluded. Some medications enhance HRV, as reported by patients^48,49^, whereas others may reduce HRV^50^ or have no significant impact^51,52^. The existing literature presents conflicting evidence on the influence of medications on HRV and cardiovascular health, with much of the limited research concentrated on animal studies or different patient demographics and healthy individuals. Additionally, smoking or nicotine intake can reduce HRV^53,54^. Four patients were reportedly nicotine users and we also cannot exclude the effect of nicotine on HRV in these patients.

No symptoms related to AD or orthostatic hypotension occurred during the high-PAS study, although they are frequent in SCI patients and might occur without overt symptoms. HRV is affected by several factors. The environment, recording time, equipment, emotional factors, pain, and fatigue play a role in HRV regulation^13,55^. Parasympathetic activation can also increase due to slow breathing^15^, which was also noted in this study (16% reduction during the stimulation compared with pre values). Additionally, the observed parasympathetic activation was moderate and did not lead to bradycardia. Five patients had low heart rate (< 60 bpm). Of these, four had low HR throughout the study and one had HR of 62 bpm at pre, which decreased to 58 bpm during stimulation and remained between 57 and 60 bpm until the end of the 60-min follow up. Bradycardia can be associated with several conditions, such as autonomic dysfunction, infections, metabolic conditions, medications, and trauma^56^. The changes in blood pressure were limited within a range of 16 mmHg (mean 2.7 mmHg).

Alertness decreased during high-PAS, similar to our previous study^36^. Many patients and healthy participants tend to sleep during high-PAS, although sleeping is not recommended during stimulation. Pain has been reported to reduce RR and increase LF power^57^. One participant reported that high-PAS was painful but had an increase in mean RR (+ 5.4%) and decrease in LF power (− 6.7%) during stimulation, indicating that the sympathetic nervous system was not activated. When initiating high-PAS with a new patient, we increase the PNS intensity gradually to facilitate adaptation^7^. However, in this single-session study, we did not have the option to modify the intensity. Additionally, this was the first session after months for the patients, leading to greater experience of stimulus-related sensation. In long-term high-PAS studies, we offer local anesthesia (5% lidocaine/prilocaine [EMLA]) cream to reduce pain^58^ for those participants who experience PNS as painful. EMLA was not used in this study, because the patients chose not to use it in their previous high-PAS treatments. For one patient, we reduced PNS intensity from the value recorded during the mapping session to highest tolerated intensity. One patient felt mild pain during the stimulation and 2 patients reported high-PAS as uncomfortable. PNS causes a pricking sensation, and a train of six pulses causes a sensation of prolonged muscle contraction. The related involuntary movement might cause unpleasant sensations. Some patients can experience tension in the neck area that can lead to a temporary tension headache due to prolonged sitting or TMS pulses; a comfortable sitting position is thus essential. TMS is usually well tolerated during high-PAS regardless of the high intensity. Some patients experience discomfort from involuntary movement that can be present in the stimulated or contralateral limb, shoulders, or in facial muscles. In this study, 7 patients had RMT level < 60% of MSO, which leads to a notable increase in stimulation intensity in relation to their motor threshold and can lead to an increased sensation of discomfort compared with participants with RMT near 100%.

MEP amplitudes increased non-significantly after high-PAS, consistent with our previous study^9^. Muscle spasticity and fatigue can possibly explain variability in MEP amplitude enhancements; in SCI patients, MEP amplitude increase is sometimes absent due to the weakened muscles, although plastic response at synaptic level may have occurred^9,10,59–63^. MEP amplitude increases have been used as one of the primary outcomes for high-PAS induced corticospinal excitability in our previous studies in heathy subjects in upper^64^ and lower limbs^59,60^. Roy et al.^65^ found increased MEP amplitude in some patients with SCI, stating that in patients with less severe injury the increase is possible, although delayed in comparison to healthy controls. We have previously tested a small group of patients with motor point integrity test,^66^ which indicated more prominent lower motor neuron injury in a patient that had low functional gain from high-PAS. Motor imagery and pre-activation enhance plasticity and are used in PAS studies^7,67^. In this study, participants were asked to focus on the targeted muscles, however, adding movement and pre-activation could have possibly improved plastic changes.

The small number of patients and lack of sham group are major limitations of this study. Additionally, the heterogeneity of the patient population, with unique injury characteristics in each patient, makes it harder to generalize the results. Adding a sham group could allow distinguishing the effects of sitting quietly from those of high-PAS. In our previous study in healthy participants, we reported a similar trend in results with 5 participants in active high-PAS and sham PAS. Additionally, HRV could have been recorded for longer periods over several days, including an orthostatic test to establish every individual’s typical profile and intra-individual variability at baseline. Moreover, HRV was recorded only during a single session of high-PAS. High-PAS therapy for SCI patients is applied over several weeks or months. Evaluation during a longer period, including several sessions of high-PAS, should be conducted in the future, as the current results cannot be generalized to cover longer intervention periods.

## Conclusion

High-PAS does not produce an acute increase in sympathetic activation in patients with incomplete cervical SCI that could be linked to pain or stress. Most changes were observed in the parasympathetic nervous system and were reversible within the 60-min follow-up period. The observed increase in parasympathetic activation was moderate; stimulation did not induce bradycardia or hypotension. The results were similar to our previous study in healthy participants^34^. It is likely that sitting still for 20 min, instead of high-PAS stimulation, plays a role in parasympathetic activation. High-intensity TMS and high-frequency PNS did not increase sympathetic (stress) activation during stimulation, although one patient found stimulation painful.

## Supplementary Information

Supplementary Information.

## Data Availability

The datasets generated during and analyzed during the current study are available from the corresponding author upon reasonable request.
