# Supplementary material for: Heart rate variability in patients with incomplete spinal cord injury during a single session of paired associative stimulation
Source: Sci Rep. 2025 Nov 25;15:41869. doi: 10.1038/s41598-025-25802-x (PMC12647663; doi:10.1038/s41598-025-25802-x)
Supplement: Supplementary file 1 — Supplementary Information. [file 41598_2025_25802_MOESM1_ESM.pdf]

Supplementary material

Supplementary Table 1. Energy and feelings before and after high-PAS. Scale 1 = very good/very comfortable, 2 = good/comfortable, 3 = not good or bad, 4 = bad/uncomfortable, 5 = very bad/very uncomfortable.

ID	Pre stim. Sleep	Sleep hours	Pre stim. Alertness	Pre stim. Stress	Alertness during PAS	Feelings during PAS	Pain during stimulation	Pain pre 24h	Activity pre 24h	Medication	Caffeine	Nicotine	Time of day
1	3	5	3	2	4	3	no	VRS 4/10	normal	normal	1 cup coffee at 7:30		10-12 morning
2	1	7	1	1	1	1	no	VRS 4/10	normal	normal	0,5 cup coffee at 6	no	9:30-11:30 morning
3	2	7	2	3	3	1	no	VRS 5/10	low	normal	no		10-12 morning
4	2	6	3	3	2	3	no	VRS 6/10	low	normal	1,5 cup coffee at 8	no	12-14 afternoon
5	2	9	1	3	3	3	no	no	normal	normal	no		14:30-16:30 afternoon
6	1	8.5	1	3	2	1	no	no	normal/high	normal	2 cups coffee at 7		9-11:15 morning
7	1	8	1	1	1	2	no	no	normal	normal	1 cup coffee at 9		11:45-13:45 afternoon
8	2	9	2	2	3	3	no	VRS 4/10	normal	normal	2 cups coffee at 6:30	yes	15-17 afternoon
9	5	4	1	2	3	4	no	no	normal	normal	1 cup coffee at 9:00	yes	14-16 afternoon
10	2	8.5	3	2	3	4	yes	VRS 2/10	normal	baclofen 5mg added a week ago	1 cup coffee at 8	yes	13-15 afternoon
11	2	6	3	2	3	3	no	no	normal	normal	1 cup coffee at 8:30	yes	15:30-17:30 afternoon
12	2	5	4	3	5	3	no	no	normal	pregabalin dose decreased 2 days ago	no	no	13:30-15:30 afternoon

Supplementary Table 2. Mean values (± SD) for different measurement time points and global test results (Friedman's test p value and Kendall's W as effect size).

Variable	Friedman's test	Effect size (Kendall's W)	PRE (mean ± SD)	STIM (mean ± SD)	POST (mean ± SD)	POST30 (mean ± SD)	POST60 (mean ± SD)
MEP (% of PRE)	0.231						
Mean RR (ms)	0.031	0.222	902.5 ± 220.86	923.71 ± 201.36	918.34 ± 215.52	939.45 ± 216.03	928.80 ± 218.22
SDNN (ms)	0.008	0.29	22.08 ± 18.03	17.12 ± 11.03	23.49 ± 15.44	23.96 ± 14.30	22.41 ± 17.77
Mean HR (bpm)	0.031	0.222	70.27 ± 17.38	68.05 ± 15.81	68.74 ± 16.25	67.11 ± 15.91	68.08 ± 16.77
SD HR (bpm)	0.002	0.346	1.52 ± 0.63	1.14 ± 0.38	1.68 ± 0.77	1.66 ± 0.68	1.52 ± 0.73
Min HR (bpm)	0.003	0.332	66.00 ± 15.77	64.72 ± 15.10	63.83 ± 14.90	62.10 ± 14.15	63.72 ± 15.75
Max HR (bpm)	0.005	0.307	76.76 ± 17.24	72.43 ± 15.86	75.25 ± 17.35	73.85 ± 16.39	74.45 ± 17.47
RMSSD (ms)	0.225		21.65 ± 22.06	18.96 ± 14.64	22.64 ± 21.13	22.08 ± 17.56	20.92 ± 19.32
LF power (ms2)	<0.001	0.399	409.42 ± 712.29	139.77 ± 156.88	376.87 ± 517.48	288.13 ± 261.34	359.68 ± 539.67
HF power (ms2)	0.274		204.55 ± 350.44	215.01 ± 326.66	242.85 ±427.35	213.40 ± 368.60	239.94 ± 511.96
LF power (n.u.)	<0.001	0.396	65.75 ± 12.58	43.86 ± 19.35	67.51 ± 11.50	60.80 ± 21.07	61.92 ± 17.23
HF power (n.u.)	<0.001	0.396	34.23 ± 12.56	56.12 ± 19.34	32.46 ± 11.49	39.15 ± 21.00	38.03 ± 17.22
Total power (ms2)	0.065		706.95 ± 1149.76	391.84 ± 497.35	691.49 ± 995.00	581.90 ± 648.76	719.13 ± 1250.81
LF-HF ratio	<0.001	0.396	2.45 ± 1.73	1.08 ± 0.98	2.45 ± 1.19	2.34 ± 1.65	2.49 ± 2.56
Resp (Hz)	0.016	0.254	0.27 ± 0.05	0.26 ± 0.05	0.22 ± 0.05	0.24 ± 0.05	0.28 ± 0.08
SD1 (ms)	0.225		15.34 ± 15.64	13.43 ± 10.37	16.04 ± 14.97	15.64 ± 12.44	14.82 ± 13.69
SD2 (ms)	<0.001	0.407	26.87 ± 20.72	19.91 ± 12.12	28.73 ± 16.70	29.72 ± 16.69	27.70 ± 21.58
SD2/SD1 ratio	0.025	0.232	2.15 ± 0.83	1.71 ± 0.73	2.21 ± 0.78	2.19 ± 0.77	2.10 ± 0.68

Supplementary table 3 Pairwise comparisons.

Variable	PRE-STIM	PRE-POST	PRE-POST30	PRE-POST60	STIM-POST	STIM-POST30	STIM-POST60	POST-POST30	POST-POST60	POST30-POST60
Mean RR (ms)	p = 1.000, +2.9%	p = 1.000, +2.06%	p = 0.67, +4.62	p = 0.201, +3.55%	p = 1.000, -0.79%	p = 1.000, +1.62%	p = 1.000, 0.49%	p = 0.282, +2.44%	p = 0.707, +1.31%	p = 1.000, -1.18%
SDNN (ms)	p = 0.528, -16.41%	p = 1.000, +17.83%	p = 1.000, +19.10%	p = 1.000, +5.03%	**p = 0.030 *, +44.59%**	**p = 0.012 *, +46.48%**	**p = 0.045 *, +29.06%**	p = 1.000, +4.86%	p = 1.000, -5.85%	p = 1.000, -7.74%
Mean HR (bpm)	p = 1.000, -2.68%	p = 1.000, -1.88%	p = 0.067, -4.04%	p = 0.201, -2.58%	p = 1.000,+0.88%	p = 1.000, -1.42%	p = 1.000, 0.00%	p = 0.282, -2.27%	p = 0.707, -0.86%	p = 1.000, +1.36%
SD HR (bpm)	P = 0.282, -21.19%	p = 0.933, +13.99%	p = 1.000, +9.58%	p = 1.000, 1.05%	**p = 0.001 **, +47.86%**	**p = 0.030 *, +42.55%**	p = 0.098, +30.48%	p = 1.000, -0.47%	p = 1.000, -7.90%	p = 1.000, -4.59%
Min HR (bpm)	p = 1.000, -1.52%	p = 0.707, -3.07%	**p = 0.005 *, -5.47%**	**p = 0.045 *, -3.07%**	p = 1.000, -1.45%	p = 0.098, -3.97%	p = 0.528, -1.65%	p = 0.933, -2.52%	p = 1.000, -0.08%	p = 1.000, +2.39%
Max HR (bpm)	**p = 0.008 *, -5.40%**	p = 1.000, -1.97%	p = 0.067, -3.41%	p = 0.389, -2.72%	p = 0.142, +3.71%	p = 1.000, +2.09%	p = 1.000, +2.83%	p = 0.707, +8.78%	p = 1.000, -0.90%	p = 1.000, +0.78%
LF power (ms2)	p = 0.142, -43.19%	p = 1.000, +64.75%	p = 1.000, +75.10%	p = 1.000, +7.81%	**p = 0.001 **, +282.77%**	p = **0.008 *, +171.66%**	**p = 0.019 *, +134.66%**	p = 1.000, +8.78%	p = 1.000, +0.98%	p = 1.000, +12.20%
LF power (n.u.)	**p = 0.008 *, -34.84%**	p = 1.000, +4.71%	p = 1.000, -7.26%	p = 1.000, -4.23%	**p = 0.002 **, +84.10%**	p = **0.005 *, +51.74%**	**p = 0.030 *, +62.43%**	p = 1.000, -10.53%	p = 1.000, -6.51%	p = 1.000, +10.06%
HF power (n.u.)	**p = 0.008 *, +70.86%**	p = 1.000, +3.32%	p = 1.000, +19.62%	p = 1.000, +16.40%	**p = 0.002 **, -36.61%**	p = **0.005 *, -30.09%**	**p = 0.030 *, -30.03%**	p = 1.000, +23.70%	p = 1.000, +29.34%	p = 1.000, +7.73%
LF-HF ratio	**p = 0.008 *, -57.05%**	p = 1.000, +20.52%	p = 1.000, +10.16%	p = 1.000, +5.10%	**p = 0.002 **, +289.01%**	p = **0.005 *, +171.81%**	**p = 0.030 *, +194.77%**	p = 1.000, -0.78%	p = 1.000, -0.06%	p = 1.000, 31.49%
Resp (Hz)	p = 1.000, -1.85%	**p = 0.019 *, -16.20%**	p = 0.528, -11.05%	p = 1.000, +6.23%	p = 0.098, -14.60%	p = 1.000, -8.95%	p = 1.000, +8.17%	p = 1.000, +7.55%	p = 0.282, +27.10%	p = 1.000, +19.70%
SD1 (ms)	p = 0.814, -0.35%	p = 0.150, +13.41%	p = 0.308, +18.00%	p = 0.347, +7.98%	p =0.272, 18.42%	**p =0.041** *, +21.56	p = 0.937, +13.61%	p = 0.347, +5.45%	p = 0.638, -2.69%	p = 0.347, -7.48%
SD2 (ms)	p = 0.707, -20.67%	p = 0.933, +19.47%	p = 0.528, +19.82%	p = 1.000, +4.53%	**p = 0.005 *, +54.73%**	**p = 0.002 **, +55.61%**	**p = 0.012 *, +34.78%**	p = 1.000, +4.75%	p = 1.000, -6.68%	p = 1.000, -7.77%
SD2/SD1 ratio	p = 0.201, -18.07%	p = 1.000, +5.56%	p = 1.000, +3.96%	p = 1.000, +2.80%	**p = 0.030 *, +35.65%**	p = 0.142.+32.70%	p = 0.098, +28.66%	p = 1.000, +0.63%	p = 1.000, -1.25%	p = 1.000, -1.39%
